# Dihydromyricetin Remodels the Tumor Immune Microenvironment in Hepatocellular Carcinoma: Development and Validation of a Prognostic Model

**DOI:** 10.3390/cimb47121010

**Published:** 2025-12-02

**Authors:** Yang Xu, Chao Gu, Wei Li, Fei Lan, Jingkun Mao, Xiao Tan, Pengfei Li

**Affiliations:** Department of Medicine, Tarim University, Alar 843300, China; xuy03262418@163.com (Y.X.); 120210122@taru.edu.cn (C.G.); dykdzy@163.com (W.L.); lf17794969636@163.com (F.L.); mjkyyx@163.com (J.M.); 13403877596@163.com (X.T.)

**Keywords:** hepatocellular carcinoma, dihydromyricetin, tumor immune microenvironment, network pharmacology, prognostic model

## Abstract

Background: Dihydromyricetin (DHM), a natural dihydroflavonol, exhibits diverse pharmacological properties, including anti-inflammatory, antioxidant, and anti-tumor effects. However, its potential mechanism of action in the individualized therapy of hepatocellular carcinoma (HCC) remains unclear. Methods: Potential therapeutic targets of DHM were identified using the Swiss Target Prediction database. The overlap between these targets and differentially expressed genes in HCC was analyzed to determine therapeutic targets. A prognostic model was constructed based on these genes, and patients were stratified into high- and low-risk groups. The associations between risk scores, clinical pathological characteristics, and overall survival were analyzed using Cox regression and Kaplan–Meier survival curves. The relationships between risk score and immune cell infiltration, immunosuppressive factors, and anticancer drug susceptibility were evaluated. Results: A three-gene prognostic model was established, comprising *DTYMK*, *MAPT*, and *UCK2*, designated as DHM-target genes (DHMGs). Patients in the high-risk group had significantly shorter overall survival than those in the low-risk group (*p* < 0.001; HR [95% CI] = 4.953 [2.544, 9.645]). Higher risk scores were correlated with more advanced tumor stages and grades. Comprehensive analysis of the tumor immune microenvironment revealed that high-risk patients exhibited significantly elevated TIDE scores, increased Treg cell infiltration, and markedly reduced stromal scores. Conclusions: This study developed a prognostic model based on the potential target genes of DHM in HCC. This model effectively stratifies HCC patients, identifying a high-risk subgroup characterized by an immunosuppressive microenvironment. These findings provide a theoretical foundation for exploring DHM as a promising natural adjuvant for cancer immunotherapy.

## 1. Introduction

Hepatocellular carcinoma (HCC) is a leading cause of cancer-related mortality worldwide, accounting for 7.8% of all cancer deaths and ranking as the third most common cause in 2022 [1]. Current standard treatments for HCC, including surgery, chemotherapy, and radiofrequency ablation, are often limited in their therapeutic efficacy. For most patients with advanced HCC, systemic precision therapies, which include targeted therapy, immunotherapy, and combination regimens, represent the optimal approach [2,3]. Consequently, there is a critical need to develop safe and effective pharmaceuticals as adjunctive or alternative treatments for HCC.

Dihydromyricetin (DHM) is a natural dihydroflavonol compound present in various plant species, including *Vitis vinifera* L., *Myrica cerifera* L., *Prunus amygdalus* Batsch, and *Ginkgo biloba* L [4]. Extensive research has demonstrated that DHM possesses a range of pharmacological properties, such as anti-inflammatory [5], antioxidant [6], anticancer [7], and hepatoprotective effects [8]. Previous studies have indicated that DHM can modulate the proliferation, migration, and apoptosis of HCC cells [7,9,10]. However, the precise mechanism of action of DHM in tailored HCC therapy remains unclear.

Despite recognition of DHM’s antitumor potential, its systemic mechanisms of action in HCC are not fully elucidated, particularly concerning its influence on the tumor immune microenvironment (TIME) and its implications for personalized therapy. Existing research primarily focuses on its direct effects on cancer cells, lacking investigations into the association between DHM-related genes and patient prognosis or the immune context.

To address this gap, our study employs an integrated framework of network pharmacology and transcriptomics to innovatively construct and validate a novel prognostic model based on DHM-associated gene signatures. In-depth analysis of the distinct immune microenvironment profiles associated with these signatures reveals their significant impact on immune responses and potential synergy with immunotherapy. Furthermore, to structurally validate the interactions between DHM and the products of key prognostic genes, we performed molecular docking analyses to evaluate the potential for direct binding. This provides structural biology support for our multi-omics findings. Ultimately, this research offers a novel perspective on DHM as a potential regulator of the TIME and proposes concrete biomarker signatures for HCC risk stratification.

## 2. Materials and Methods

### 2.1. Datasets

Transcriptomic and clinical data for HCC were obtained from two primary sources. From The Cancer Genome Atlas (TCGA) Liver Hepatocellular Carcinoma (TCGA-LIHC) project, we retrieved high-throughput RNA sequencing (RNA-seq) data and corresponding clinical information for 374 tumor samples and 50 adjacent normal tissue samples [11]. Furthermore, RNA-seq data and clinical records for the HCCDB18 cohort (ICGC-LIRI-JP), comprising 212 tumor and 177 normal control samples, were acquired via the HCCDB 2.0 database [12]. Additionally, the gene expression datasets GSE109211 [13] and GSE94550 [14] were obtained from the Gene Expression Omnibus (GEO) to identify molecular features of sorafenib resistance.

### 2.2. Data Preprocessing and Standardization

Data from the TCGA-LIHC cohort were processed in two formats: raw counts and transcripts per million (TPM). Raw counts were used to identify DEGs, while TPM values were utilized for immune cell deconvolution with the CIBERSORT package and for drug sensitivity analysis. For all other analyses, a log_2_(TPM + 1) transformation was applied. After excluding samples with incomplete survival data, 365 tumor samples were retained for subsequent analysis. The mean and standard deviation of gene expression were calculated from the TCGA-LIHC TPM data (Table 1), and Z-score standardization was performed to construct the LASSO model. For the independent HCCDB18 dataset, expression data were uniformly converted to TPM format and then standardized using the mean and standard deviation derived from the TCGA-LIHC cohort to ensure consistency for risk score calculation and CIBERSORT analysis. For the sorafenib resistance analysis, the GSE109211 and GSE94550 datasets were filtered to include only sorafenib-treated patients, resulting in 67 samples (46 sensitive, 21 resistant) and 9 samples (3 sensitive, 6 resistant), respectively.

### 2.3. DHM-Related Target Acquisition

The canonical SMILES structure of DHM was obtained from the PubChem database [15]. Potential molecular targets of DHM were identified using the Swiss Target Prediction [16] and PharmMapper databases [17]. Targets from Swiss Target Prediction were filtered with “Probability > 0.1”. Those from PharmMapper were standardized using UniProt [18] to ensure uniform gene nomenclature. The resulting lists were consolidated and deduplicated to generate a final set of unique DHM-related targets.

### 2.4. HCC Differential Gene Screening

Identified DEGs between tumor and adjacent non-tumor tissues in the TCGA-LIHC dataset using the R package ‘limma’ (version 3.62.2), applying thresholds of |log_2_ fold change (FC)| > 1 and FDR < 0.001.

### 2.5. Prognostic Gene Modeling

The intersection between DHM-related targets and HCC-related DEGs was identified using the R package ‘ggvenn’ (version 0.1.16). Genes from this intersection that demonstrated prognostic significance (*p* < 0.01) were selected via univariate Cox regression analysis of the TCGA-LIHC dataset. A prognostic risk model was then constructed using least absolute shrinkage and selection operator (LASSO) Cox regression. To ensure model robustness, we performed 100 iterations of 10-fold cross-validation with a fixed random seed (123456). The risk score for each patient was calculated using the following formula:

$$Risk\;score=\sum_{i=1}^{n}\frac{{expression}_{gene}-{Mean}_{gene}}{{Std}_{gene}}\times {coefficients}_{gene}$$

Based on the median risk score, HCC patients were stratified into high-risk and low-risk groups. Time-dependent ROC analysis was performed on the TCGA clinical data using the R packages ‘timeROC’ (version 0.4) and ‘survminer’ (version 0.5.0). Similarly, survival analysis between the risk groups was conducted using the ‘survival’ (version 3.5-8) and ‘survminer’ packages. Finally, the model was validated in the independent HCCDB18 dataset through both survival analysis and time-dependent ROC analysis.

### 2.6. Construction of Nomogram

To ensure cohort integrity, we excluded TCGA-LIHC samples with missing clinicopathological data (such as grade, stage, or TNM classification), resulting in a final analytical cohort of 229 patients. The association between risk scores and specific clinicopathological features was assessed using the Wilcoxon rank-sum test. To determine the independent prognostic value of the genetic signature, we performed univariate and multivariate Cox regression analyses, adjusting for clinical parameters including age, gender, overall stage, grade, and TNM stage. A predictive nomogram integrating the risk score with significant clinical covariates was subsequently developed. The model’s predictive accuracy for overall survival at multiple time points was evaluated using time-dependent ROC curves.

### 2.7. Immune Infiltration Analysis

The anti-tumor immune response, known as the cancer-immunity cycle, involves a sequential process from cancer antigen release to immune-mediated killing of cancer cells [19]. This framework underpins immune-mediated tumor recognition and elimination, which directly influences the efficacy of immunotherapy.

To compare anti-tumor immunity across risk groups, we obtained gene sets representing each step of the cycle from the Tracking Tumor Immunophenotype (TIP) database and calculated corresponding activity scores. We next characterized the TIME using transcriptomic data from the TCGA-LIHC and HCCDB18 cohorts.

For immune cell composition, we employed two complementary approaches. First, we estimated the relative proportions of 22 immune cell subtypes using the CIBERSORT algorithm. Second, we performed GSVA using feature gene sets for 28 immune cell types from the TISIDB database to calculate enrichment scores as a proxy for relative cell abundance [20] (Table S12).

To assess immune functional activity, we applied GSVA to 13 immune-related functional gene sets from He et al. [21]. Potential immunotherapy response was evaluated by uploading log_2_-transformed gene expression data to the Tumor Immune Dysfunction and Exclusion (TIDE) platform to compute TIDE scores [22]. Finally, we analyzed correlations between risk scores and specific immunosuppressive molecules using the TISIDB database.

### 2.8. Drug Sensitivity Analysis

To predict chemotherapeutic drug sensitivity across risk groups, we utilized the Genomics of GDSC database [23] and applied the R package oncoPredict (version 1.2). Using the GDSC2 reference set, we estimated the half-maximal inhibitory concentration (IC_50_) with parameters set to batchCorrect = ‘eb’ and a random seed of 999.

Separately, we identified Sorafenib resistance gene (SRGs) from the GSE109211 and GSE94550 datasets using the R package limma, applying thresholds of |log_2_(fold change)| > 1 and FDR < 0.01. The overlap between SRGs from both datasets was defined as the core sorafenib sensitivity signature.

We integrated four training datasets, including TCGA-LIHC, HCCDB6 (GEO14520), HCCDB18, and HCCDB25 (OEP000321), and removed batch effects using the ComBat algorithm. The R package caret was then employed to perform stratified sampling by survival status, randomly allocating 70% of the tumor samples to a training set and 30% to a testing set.

To construct a robust predictive model, we integrated ten machine learning algorithms: Random Survival Forest (RSF), LASSO, Survival-SVM, GBM, SuperPC, CoxBoost, Ridge Regression, plsRcox, StepCox, and Elastic Net (Enet). We evaluated 101 variable selection combinations across these algorithms to build sorafenib sensitivity features. To identify the optimal combination, we calculated the C-index for each model across all cohorts [24].

### 2.9. Molecular Docking

To evaluate the binding affinity and interaction modes between DHM and its potential targets, we performed molecular docking using AutoDock Vina (v.1.1.2) and AutoDockTools (v.1.5.7) [25]. The three-dimensional structure of DHM was retrieved from the PubChem database, and the crystal structures of the target proteins were obtained from the Protein Data Bank (PDB) [26]. Protein and ligand files were prepared by converting them to PDBQT format, removing water molecules, and adding polar hydrogen atoms. Docking simulations were executed with the exhaustiveness parameter set to 10. The resulting binding poses and interaction patterns were visualized and analyzed using PyMOL (v.3.1.3.1) and LigPlus (v.2.2.9).

### 2.10. Statistical Analysis

All statistical analyses were conducted using R software (version 4.3.3). The specific versions of all R packages used are detailed in Table S13. Intergroup comparisons were performed using the Wilcoxon rank-sum test, and correlation analyses were assessed using Spearman’s rank correlation coefficient. A *p*-value of less than 0.05 was considered statistically significant.

## 3. Results

### 3.1. Screening for DHM-Associated Genes Relevant to Prognosis

**Figure 1:**
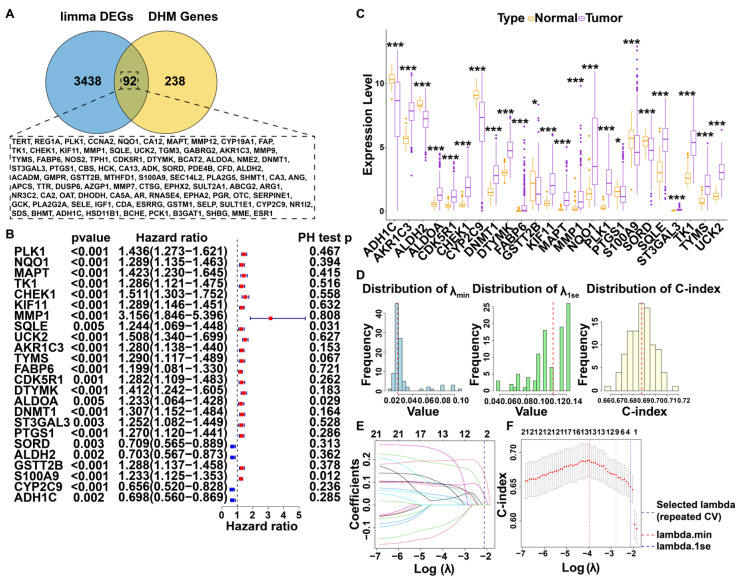
Identification of potential therapeutic targets for DHM in HCC and construction of prognostic models. (**A**) Genes at the intersection of DHM potential targets and DEGs. (**B**) Differential expression of 24 therapeutic targets in tumor and normal samples. (**C**) Univariate Cox regression analysis. (**D**) Distribution plot of LASSO model validation results from 100 replicates. (**E**) LASSO regression coefficient path plot. (**F**) LASSO regression C-index distribution plot (* *p* < 0.05, *** *p* < 0.001).

The Swiss Target Prediction database predicted 100 potential targets for DHM, which were filtered to 70 targets using a probability criterion of >0.1 (Table S1). Separately, the PharmMapper database predicted 299 targets (Table S2). The integration of results from both databases yielded 330 unique DHM-related target genes (Table S3). Meanwhile, differential expression analysis performed with the R package “limma” identified 3530 differentially expressed genes (DEGs) in HCC, using the thresholds of |log_2_ fold change| > 1 and false discovery rate (FDR) threshold of <0.001 (Table S4). A cross-referencing of these DEGs with the 330 DHM-related targets revealed 92 overlapping genes, which were defined as DHM-related targets for further analysis (Figure 1A, Table S5).

### 3.2. Construction and Evaluation of Prognostic Models Based on DHM-Related Targets

Among the 92 identified DHM-associated differentially expressed targets, 24 were significantly correlated with patient overall survival (OS). A Schoenfeld Residuals Test was performed for these 24 genes (Figure 1B and Figure S1), all of which demonstrated significant differential expression between tumor and normal tissue (*p* < 0.05; Figure 1C). Following 100 repetitions of 10-fold cross-validation, the LASSO regression analysis yielded median values of 0.019 for lambda.min, 0.1197 for lambda.1se, and 0.686 for the concordance index (C-index) (Figure 1D). Using lambda.1se (value = 0.1197) for model construction, the DHM-related targets were refined to three genes via LASSO Cox regression: *DTYMK*, *MAPT*, and *UCK2* (Figure 1E). The resulting model had a C-index of 0.679 (Figure 1F). These three genes were designated as DHM-target genes (DHMGs). Survival analysis revealed that patients with high expression of any of the three DHMGs had a significantly poorer prognosis, with hazard ratios as follows: *DTYMK* (HR = 2.240, 95% CI [1.586–3.165]; Figure S2A), *MAPT* (HR = 1.707, 95% CI [1.208–2.411]; Figure S2B), and *UCK2* (HR = 2.217, 95% CI [1.565–3.140]; Figure S2C).

### 3.3. Survival Analysis and Nomogram Construction

**Figure 2:**
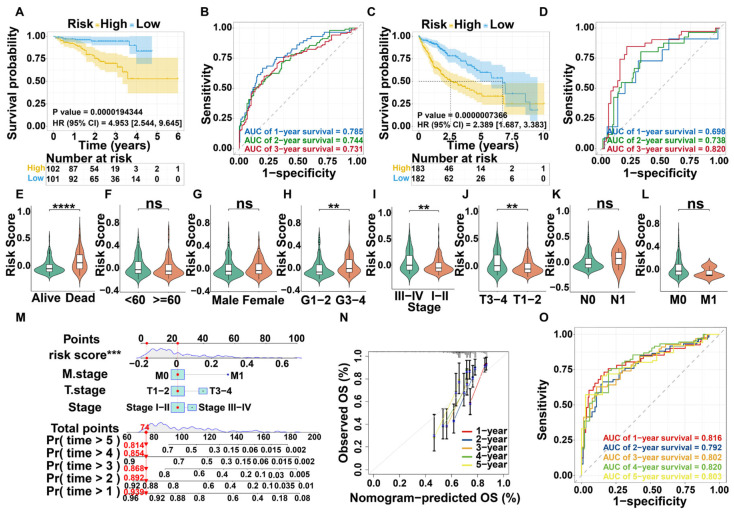
Prognostic survival analysis and nomogram construction. (**A**) Survival analysis of TCGA-LIHC. (**B**) ROC curve of TCGA-LIHC. (**C**) Survival analysis of HCCBD18. (**D**) Survival analysis of HCCDB18. (**E**) OS events. (**F**) Age. (**G**) Sex. (**H**) Histologic grading. (**I**) Pathologic staging. (**J**) T-staging. (**K**) N-staging. (**L**) M staging. (**M**) Nomogram of multivariate analysis based on risk scores and clinical characteristics. (**N**) Calibration plot showing the predictive accuracy of the model. (**O**) Time-dependent ROC curves. (** *p* < 0.01, *** *p* < 0.001, **** *p* < 0.0001, and ns, no statistical difference).

HCC patients from the TCGA-LIHC cohort were stratified into high- and low-risk groups based on the median risk score derived from the DHMG signature (Figure S2D, Table S6). Subsequent survival analysis revealed a significantly poorer overall survival rate in the high-risk group compared to the low-risk group (Figure 2A). The predictive accuracy of the model was further evaluated using time-dependent receiver operating characteristic (ROC) curves, which showed area under the curve (AUC) values of 0.785, 0.744, and 0.731 for 1, 2, and 3 years, respectively (Figure 2B). These results demonstrate that the DHMG signature has strong prognostic performance. Consistent with the risk stratification, the expression of all three DHMGs was significantly upregulated in the high-risk group, as visualized in a heatmap (Figure S2E).

### 3.4. Validation of Prognostic Model for DHMGs

To validate the prognostic model, the HCCDB18 dataset was stratified into high- and low-risk groups using the calculated risk scores (Figure S2F, Table S6). Survival analysis confirmed a significantly lower survival rate in the high-risk group compared to the low-risk group (Figure 2C). Furthermore, time-dependent ROC analysis demonstrated strong predictive accuracy, with AUC values of 0.698, 0.738, and 0.820 for 1, 2, and 3 years, respectively (Figure 2D). These results indicate that the DHMGs model in the validation set exhibited high accuracy in predicting survival outcomes. Consistent with the HCCDB18 cohort, a heatmap visualization showed significant upregulation of all three DHMGs in the high-risk group (Figure S2G). These results collectively validate the DHMG signature as a robust and accurate prognostic tool for HCC.

### 3.5. Nomogram Construction

The association between clinical characteristics and risk groups were assessed using the Wilcoxon signed-rank test (Table 2). Significant differences were observed between the risk groups in pathological stage, T stage, and histological grade, but not in gender, N stage, or M stage. Furthermore, risk scores were significantly higher in patients who died, confirming the risk score as an independent prognostic factor (*p* < 0.05; Figure 2E–L). Univariate Cox analysis demonstrated that risk score and several clinical characteristics, including particularly stage (HR (95% CI) = 2.974 (1.874–4.720), *p* < 0.001), T stage (HR (95% CI) = 2.991 (1.884–4.749), *p* < 0.001), M stage (HR (95% CI) = 4.055 (1.270–12.947), *p* = 0.0363), and the risk score itself (HR (95% CI) = 57.037 (16.725–194.506), *p* < 0.001), were significantly associated with prognosis in HCC patients. After adjusting for potential confounders, multivariate Cox analysis confirmed that the risk score (HR (95% CI) = 41.050 (11.493–146.614); *p* < 0.001) remained an independent prognostic factor (Table 3). A nomogram was then constructed by integrating T stage, M stage, stage, and the risk score to predict the probability of 1-, 2-, 3-, 4-, and 5-year overall survival in HCC patients (Figure 2M). The calibration curve for the nomogram indicated strong concordance between predicted and observed outcomes (Figure 2N). The predictive accuracy, evaluated by the AUC, was 0.816, 0.792, 0.802, 0.820, and 0.803 for 1 to 5 years, respectively (Figure 2O). These results indicate that the nomogram provides superior prognostic performance compared to the risk score alone, offering a more precise tool for estimating patient survival probability.

### 3.6. Immunization Landscapes of Different Risk Groups

#### 3.6.1. Immune Cell Infiltration

**Figure 3:**
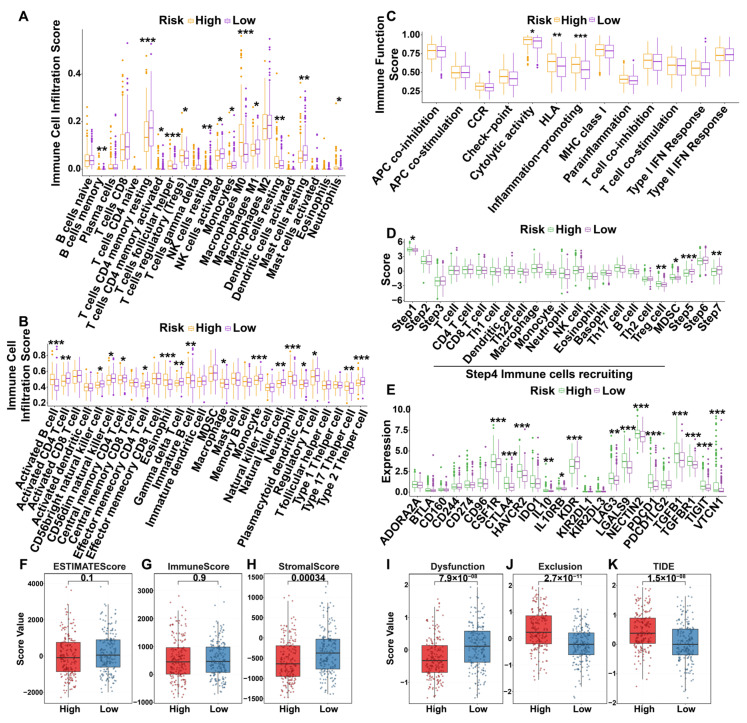
Differences in immune infiltration and function across risk groups in TCGA-LIHC. (**A**) Differential immune cell infiltration in different risk groups (CIBERSORT). (**B**) Differential immune cell infiltration in different risk groups (GSVA). (**C**) Differential immune function in different risk groups. (**D**) Differential analysis of high and low risk groups with respect to the seven steps of the cancer immune cycle. The status of anticancer immunity across seven-step Cancer-Immunity Cycle including release of cancer cell antigens (Step 1), cancer antigen presentation (Step 2), priming and activation (Step 3), trafficking of immune cells to tumors (Step 4), infiltration of immune cells into tumors (Step 5), recognition of cancer cells by T cells (Step 6) and killing of cancer cells (Step 7). (**E**) Differential expression of immunoinhibitors among different risk groups. (**F**–**H**) Relationship between different risk groups and estimate score (**F**), stromal score (**G**), and immune scores (**H**). (**I**–**K**) Relationship between different risk groups and TIDE score. (**I**) T-cell dysfunction score. (**J**) T-cell exclusion score. (**K**) TIDE score. (* *p* < 0.05, ** *p* < 0.01, *** *p* < 0.001).

To investigate systematic differences in the TIME between high- and low-risk groups, we performed a comprehensive immune infiltration analysis using transcriptomic data from the TCGA-LIHC cohort. Analyses using both CIBERSORT and gene set variation analysis (GSVA) revealed fundamental disparities in immune cell infiltration patterns between the two groups (Tables S7 and S8). Specifically, the high-risk group exhibited a pronounced immunosuppressive phenotype. CIBERSORT analysis indicated significantly elevated infiltration of immunosuppressive regulatory T cells (Tregs) and M0 macrophages, coupled with a marked reduction in anti-tumor effector cells, such as natural killer (NK) cells and CD4+ T cells (*p* < 0.05; Figure 3A). This trend was corroborated by GSVA, which showed significantly lower enrichment scores for cytotoxic immune cells, including gamma delta T cells and NK cells, in the high-risk group (Figure 3B).

To assess immune functional activity, we applied GSVA to 13 immune-related functional gene sets defined by He et al. (Table S9). The results demonstrated significant differences between risk groups in several key functions, including cytolytic activity, HLA expression, and inflammation promotion (*p* < 0.05; Figure 3C). Collectively, these multifaceted analyses reveal that the TIME of high-risk patients is characterized by a dysfunctional, immunosuppressive state. This state defined by an enrichment of immunosuppressive cells and a concomitant depletion of effector immune cells provides a compelling immunological explanation for the poor prognosis observed in these patients.

#### 3.6.2. The Seven Steps of the Anti-Tumor Immune Cycle

The genes associated with the seven phases of the anti-tumor immune cycle were retrieved from the TIP database and analyzed. The capacity for cancer antigen presentation (Step 1) was significantly elevated in the high-risk group compared to the low-risk group. In contrast, no significant differences were observed in antigen presentation or the priming and activation of immune cells (Steps 2 and 3). The chemotactic recruitment of Tregs and myeloid-derived suppressor cells (MDSCs) was significantly enhanced in the high-risk group. Furthermore, the trafficking of immune cells into tumors (Step 5) and the killing of cancer cells (Step 7) were significantly impaired in the high-risk group. However, no significant difference was found in the recognition of cancer cells by T cells (Step 6) (Figure 3D). These results suggest that HCC may persist and proliferate by exploiting immune checkpoints to evade immune surveillance.

#### 3.6.3. Immune-Related Molecules

To further characterize the immune microenvironment of different HCC risk groups, we analyzed their association with key immunological parameters using the TISIDB database. We observed significant differential expression of numerous immunostimulators, immunoinhibitors, MHC molecules, chemokines, and chemokine receptors between the groups (Figure 3E and Figure S3A–D). Subsequent correlation analysis identified the specific molecules most strongly associated with the risk score. The top correlated immunoinhibitors were *LGALS9* (R = 0.37, *p* < 0.001), *TGFB1* (R = 0.35, *p* < 0.001), *TGFBR1* (R = 0.34, *p* < 0.001), *CTLA4* (R = 0.29, *p* < 0.001), and *NECTIN2* (R = 0.29, *p* < 0.001) (Figure S4A). Similarly, the immunostimulators *CD276* (R = 0.55, *p* < 0.001), *TNFRSF4* (R = 0.40, *p* < 0.001), *TNFSF4* (R = 0.38, *p* < 0.001), *MICB* (R = 0.37, *p* < 0.001), and *TNFSF15* (R = 0.36, *p* < 0.001) showed strong positive correlations (Figure S4B). We also found significant correlations for MHC molecules (*TAP1*, *TAPBP*, *TAP2*, *HLA-DMB*, *HLA-DQA1*; Figure S4C), chemokines (*CCL20*, *CXCL3*, *CXCL5*, *CCL16*, *CXCL8*; Figure S4D), and chemokine receptors (*CCR10*, *CCR3*, *CCR8*, *CXCR4*, *CXCR3*; Figure S4E). All correlations were statistically significant (*p* < 0.001).

#### 3.6.4. Immune-Related Scores

Furthermore, the stromal score was significantly lower in the high-risk group compared to the low-risk group, although no significant differences were observed in the ESTIMATE or immune scores (Figure 3F–H). Assessment of immunotherapy efficacy potential showed that the high-risk group had a lower T-cell dysfunction score (Figure 3I) but higher TIDE and T-cell exclusion scores (Figure 3J,K). These scores also differed significantly from the Microsatellite Instability Expression Signature (MSI Expr Sig) (Figure S5A), suggesting that high-risk patients are less likely to respond favorably to immunotherapy. Additionally, key biomarkers of immunotherapy response, including Merck 18, CD274 (PD-L1), CD8, IFNG, MDSC, and CAF, were significantly differentially expressed between the risk groups (Figure S5B–H). Collectively, these findings indicate that the DHMGs play a multifaceted role in modulating the immunosuppressive landscape of HCC.

#### 3.6.5. Immunization Landscapes of Validation Dataset

**Figure 4:**
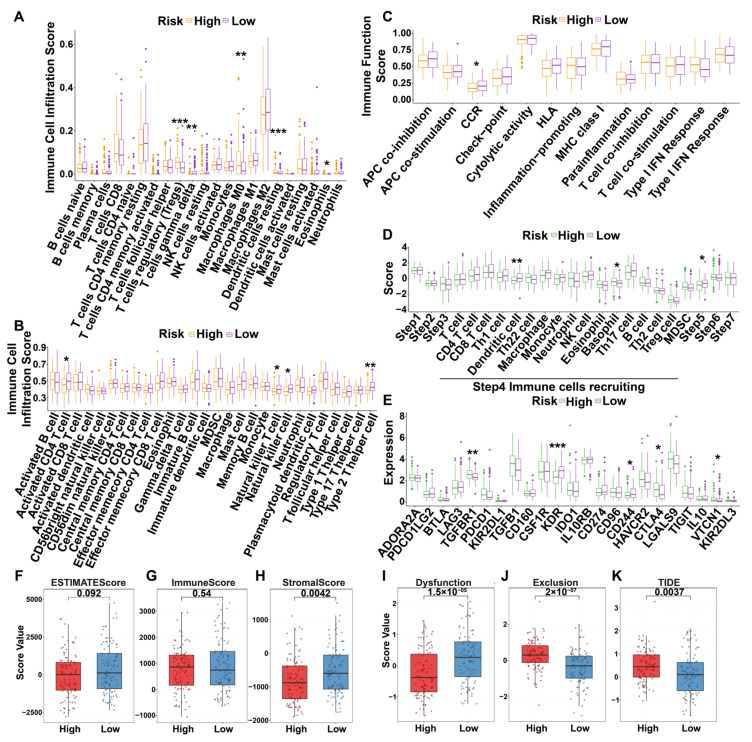
Differences in immune infiltration and function across risk groups in HCCDB18. (**A**) Differential immune cell infiltration in different risk groups (CIBERSORT). (**B**) Differential immune cell infiltration in different risk groups (GSVA). (**C**) Differential immune function in different risk groups. (**D**) Differential analysis of high and low risk groups with respect to the seven steps of the cancer immune cycle. The status of anticancer immunity across seven-step Cancer-Immunity Cycle including release of cancer cell antigens (Step 1), cancer antigen presentation (Step 2), priming and activation (Step 3), trafficking of immune cells to tumors (Step 4), infiltration of immune cells into tumors (Step 5), recognition of cancer cells by T cells (Step 6) and killing of cancer cells (Step 7). (**E**) Differential expression of immunoinhibitors among different risk groups. (**F**–**H**) Relationship between different risk groups and estimate score (**F**), stromal score (**G**), and immune scores (**H**). (**I**–**K**) Relationship between different risk groups and TIDE score. (**I**) T-cell dysfunction score. (**J**) T-cell exclusion score. (**K**) TIDE score. (* *p* < 0.05, ** *p* < 0.01, *** *p* < 0.001).

Analysis of the validation cohort confirmed a distinct immune infiltration profile in high-risk patients, characterized by elevated levels of Tregs and M0 macrophages, but reduced infiltration of CD4+ T cells, gamma delta T (γδ T) cells, and natural killer T (NKT) cells (Figure 4A,B). Furthermore, the high-risk group demonstrated a significantly impaired capacity for chemokine-cytokine receptor (CCR) interactions (Figure 4C). This was accompanied by markedly diminished recruitment of dendritic cells and basophils, as well as a reduced overall capacity for immune cell trafficking into tumors (Figure 4D). Significant differences were also observed in the expression of key immunoinhibitors, including *TGFBR1*, *CTLA4*, and *KDR*, which were elevated in the high-risk group (Figure 4E). Tumor microenvironment analysis revealed that both stromal and ESTIMATE scores were significantly lower in the high-risk group; however, the risk score correlated significantly only with the stromal score (Figure 4F). Finally, high-risk patients exhibited elevated TIDE scores and T-cell exclusion rates, alongside reduced T-cell dysfunction scores (Figure 4G), a profile indicative of a potential poor response to immunotherapy.

### 3.7. Drug Sensitivity Analysis

**Figure 5:**
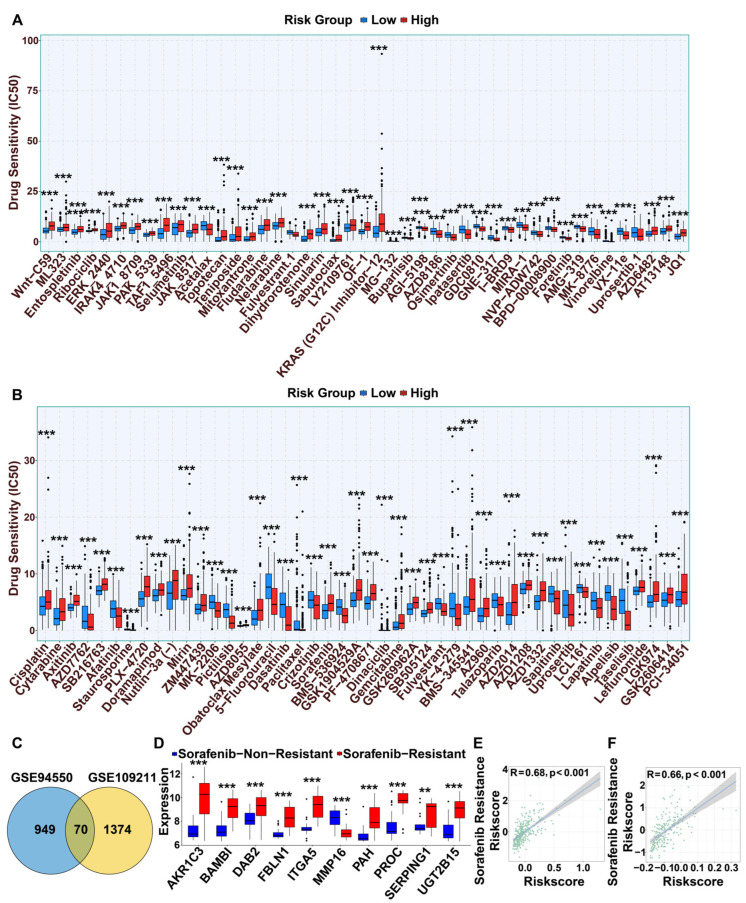
Assessment of drug sensitivity between high and low risk groups (**A**,**B**). (**C**) Screening of genes associated with sorafenib resistance. (**D**) Expression of sorafenib resistance genes in the GSE109211 dataset. (**E**) Correlation of risk scores with sorafenib resistance scores in TCGA-LIHC. (**F**) Correlation of risk scores with sorafenib resistance scores in HCCDB18. (** *p* < 0.01, *** *p* < 0.001).

Beyond immunotherapy, chemotherapy and targeted agents are cornerstone treatments for HCC. To identify therapeutics with differential efficacy, we analyzed drug sensitivity patterns across risk groups using the GDSC database, applying FDR < 0.001 for screening (Table S10). High-risk patients demonstrated significantly reduced sensitivity to most drugs, including sorafenib, axitinib, cisplatin, and selumetinib. Conversely, notable exceptions included osimertinib and lapatinib, which showed greater efficacy in the high-risk group (Figure 5A,B). These findings highlight that chemotherapeutic efficacy varies substantially between risk stratifications.

To investigate the mechanisms of sorafenib resistance, we identified sorafenib resistance-associated genes (SRGs) from the GSE109211 and GSE94550 datasets, employing thresholds of |log_2_FC| > 1 and FDR < 0.01. This analysis yielded 1444 and 1019 genes, respectively. The intersection of these gene sets revealed 70 core SRGs (Figure 5C, Table S11). A sorafenib sensitivity score was then developed by integrating 101 combinations of 10 classical algorithms. The StepCox [forward] + GBM combination was selected as the optimal model based on its superior and consistent predictive performance across all cohorts, as measured by the C-index (Figure S6A). The C-index and ROC curves for this model are shown in Figure S6B–E. This model identified nine key feature genes: *AKR1C3*, *BAMBI*, *ITGA5*, *DAB2*, *FLNC*, *CLDN1*, *SPINK1*, *PSAT1*, and *FKBP5* (Figure 5D and Figure S6F,G).

A strong positive correlation was observed between the sorafenib resistance score and the DHMG risk score in the primary cohort (R = 0.68, *p* < 0.001; Figure 5E), a finding validated in an independent dataset (R = 0.66, *p* < 0.001; Figure 5F). Among the feature genes, *AKR1C3* (R = 0.51), *BAMBI* (R = 0.45), and *ITGA5* (R = 0.42) exhibited the strongest positive correlations with the risk score (all *p* < 0.001). *DAB2*, *FBLN1*, and *MMP16* were also positively correlated. In contrast, *SERPING1*, *UGT2B15*, *PAH*, and *PROC* showed significant negative correlations (Figure S7). These results provide a foundation for personalizing treatment strategies for HCC patients.

### 3.8. Molecular Docking Validation

**Figure 6:**
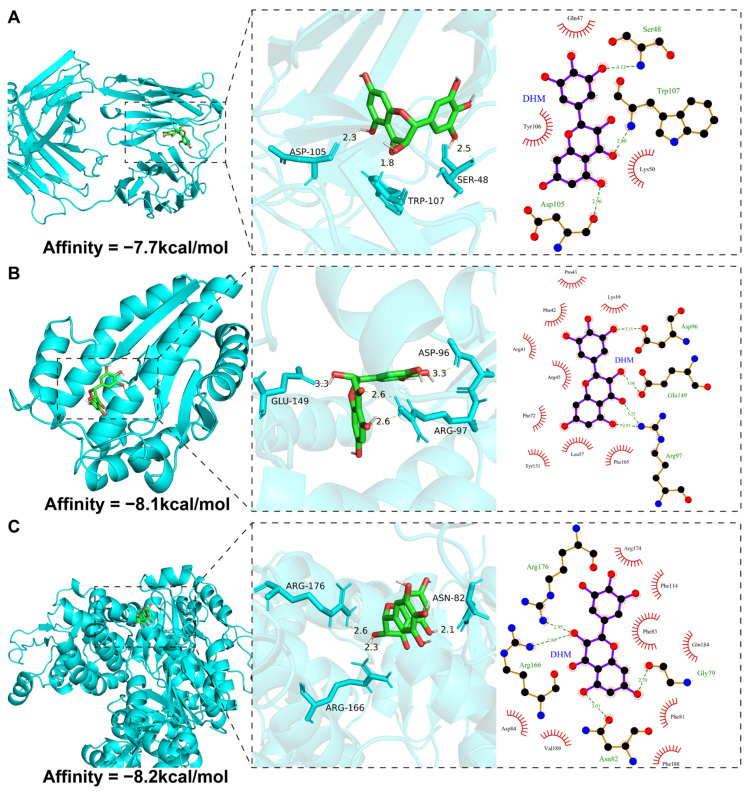
Molecular docking results of DHM with 3 DHMGs. (**A**) MAPT, (**B**) DTYMK, (**C**) UCK2.

Molecular docking was performed to assess the binding affinity between DHM and its potential target proteins. Using AutoDock Vina (v.1.1.2) and AutoDockTools (v.1.5.7), we simulated the binding conformations and interaction modes of DHM with seven proteins and calculated the corresponding binding energies (Table 4). Based on established criteria, a binding affinity more negative than −4.25 kcal/mol indicates a standard interaction, below −5.0 kcal/mol suggests good binding, and below −7.0 kcal/mol signifies strong binding activity [27]. The results demonstrated that DHM forms stable complexes with three proteins, mediated by conventional hydrogen bonds and significant electrostatic interactions (Figure 6A–C). Notably, the docking of DHM with UCK2 (PDB ID: 7SQL) yielded a highly favorable binding energy of −8.2 kcal/mol, suggesting a potential interaction.

## 4. Discussion

Previous studies have established the anti-tumor effects of DHM in various malignancies, including multiple myeloma [28], lung cancer [29], colorectal cancer [30], breast cancer [31], and bile duct cancer [32]. However, its mechanism of action in HCC remains inadequately characterized. Given the intricacy of DHM’s anti-tumor mechanisms, which likely involve multi-target and multi-pathway regulation, an exclusive focus on isolated signaling pathways is insufficient for a comprehensive understanding. Therefore, this study employed an integrated network pharmacology and transcriptomics approach to systematically elucidate the therapeutic potential of DHM in HCC and to inform personalized treatment strategies.

This study successfully establishes a link between the pharmacological activity of DHM and the clinical prognosis of HCC. We developed a robust three-gene prognostic signature (*DTYMK*, *MAPT*, and *UCK2*) by integrating network pharmacology with transcriptomic data. Each of these genes plays a pivotal role in cancer progression. DTYMK catalyzes the phosphorylation of dTMP to form dTDP, a crucial building block for DNA synthesis, and its upregulation in HCC meets the heightened demands of proliferating cells [33]. Although MAPT is predominantly expressed in neurons, its overexpression in HCC has been shown to inhibit autophagosome-lysosome fusion, thereby promoting tumor progression [34]. UCK2, the rate-limiting enzyme in the pyrimidine salvage pathway, catalyzes the phosphorylation of uridine and cytidine to support DNA and RNA synthesis; its upregulation in HCC frequently portends a poor prognosis [35]. Crucially, the prognostic risk score derived from these genes demonstrated independent prognostic value after adjusting for key clinical variables, underscoring its potential as a reliable biomarker for the personalized management of HCC.

Our multi-omics analysis identified *DTYMK*, *MAPT*, and *UCK2* as a synergistic prognostic signature. To provide a structural basis for these findings, we performed molecular docking simulations. The results yielded favorable binding energies between DHM and these proteins, with a particularly strong predicted affinity for *UCK2* (−8.2 kcal/mol). While these computational findings suggest that the prognostically significant genes may be direct structural targets of DHM, it is critical to emphasize that docking energies indicate theoretically feasible binding rather than confirmed interaction. These results thus add a dimension of plausibility to the hypothesis that DHM influences HCC progression by modulating the expression or function of these genes, a notion that requires rigorous experimental validation.

Herbal medicine demonstrates anti-tumor effects via multi-target and multi-pathway regulatory mechanisms [36]. It directly inhibits tumor cell proliferation and induces apoptosis while also regulating the immune microenvironment and suppressing inflammation [37,38]. DHM, an active component of the traditional Chinese herbal medicine Vine tea [39], similarly regulates the immune microenvironment. Our findings indicate that the adverse prognosis in the high-risk group is associated with a profoundly immunosuppressive TIME. These patients exhibit an “immune-excluded” phenotype, characterized by enhanced infiltration of Tregs and M0 macrophages, which can promote tumor growth [40,41], alongside significantly reduced infiltration of CD4+ T cells and natural killer (NK) cells. Furthermore, T-cell recruitment and cytolytic activity were markedly impaired, with dominant expression of immunosuppressive molecules. Critically, elevated TIDE and T-cell exclusion scores, coupled with a lower MSI signature, collectively depict a microenvironment where T-cells cannot effectively infiltrate and attack tumor cells. We therefore hypothesize that the DHM-associated genetic signature promotes HCC aggressiveness by fostering an immunosuppressive TIME, which aligns with DHM’s known immunomodulatory properties [42,43], though this causal relationship warrants experimental confirmation. Given that DHM often exhibits more potent antitumor effects in combination with other agents [44,45,46,47], we analyzed chemotherapeutic agents from the GDSC database to explore its combinatorial potential. Sorafenib, a first-line treatment for advanced HCC, yields a favorable response in only approximately 30% of patients [48]. The results indicate that sorafenib resistance is higher in HCC patients with elevated risk scores, accompanied by increased expression of most identified SRGs. This resistance gene profile proposes that DHM co-administration could theoretically overcome certain resistance mechanisms; however, this putative synergistic effect must be validated experimentally.

Despite the promising associations revealed by our model, the translational pathway for DHM, particularly in combination therapy, faces pharmacological challenges. A key consideration is the pharmacokinetic (PK) profile of DHM. As a natural flavonoid, it exhibits low solubility, permeability, and oral bioavailability, which may impede the attainment of effective therapeutic concentrations at tumor sites [49]. Future studies are imperative to determine the optimal dosage, schedule, and formulation strategies to improve its PK properties. Furthermore, the pharmacodynamic (PD) interactions between DHM and established immunotherapies or targeted agents remain speculative and require rigorous validation in preclinical models to assess synergy, antagonism, and potential toxicity.

This study has several limitations. First, the analyses relied exclusively on data from public databases. While valuable, such data may contain inherent discrepancies that introduce systematic bias. Future validation with larger, prospectively collected clinical cohorts and fundamental experiments is essential. Second, the identification of DHM’s targets depended on computational predictions, and the interactions suggested by molecular docking require confirmation through biochemical and cellular binding assays. Third, this work was confined to bioinformatics analyses; consequently, in vitro and in vivo functional studies are necessary to comprehensively elucidate the causal mechanisms by which DHM treats HCC.

## 5. Conclusions

This study successfully identified potential therapeutic targets of DHM in HCC by integrating network pharmacology and molecular docking, providing a rational framework for future research and drug development. We established and validated a novel prognostic signature based on three DHM-related genes (*DTYMK*, *MAPT*, and *UCK2*), which demonstrated independent predictive value for HCC patient outcomes. This model offers a promising tool for personalizing clinical management strategies for HCC. Furthermore, we propose a novel therapeutic approach combining sorafenib with DHM, which warrants further experimental investigation to validate its clinical feasibility.

## Figures and Tables

**Table 1:** Mean, Standard Deviation and Coefficient of Feature Genes Calculated Using the TCGA Dataset.

Gene	Mean	Std	Coefficient
*DTYMK*	33.960534	23.7597	0.0439839051614121
*MAPT*	1.609325	9.508439	0.024687244385511
*UCK2*	9.508439	7.874771	0.134099098314701

**Table 2:** Relationship between risk group and clinicopathologic features of HCC patients.

Characteristic	High Risk	Low Risk	*p* Value
*n*	115	114	
Age, *n* (%)			0.262
<60	65 (56.5%)	55 (48.2%)	
<60	50 (43.5%)	59 (51.8%)	
Gender, *n* (%)			0.505
Female	39 (33.9%)	33 (28.9%)	
Male	76 (66.1%)	81 (71.1%)	
T stage, *n* (%)			0.03
T1–2	75 (65.2%)	90 (78.9%)	
T3–4	40 (34.8%)	24 (21.1%)	
N stage, *n* (%)			0.622
N0	112 (97.4%)	113 (99.1%)	
N1	3 (2.61%)	1 (0.88%)	
M stage, *n* (%)			0.622
M0	114 (99.1%)	112 (98.2%)	
M1	1 (0.87%)	2 (1.75%)	
Stage, *n* (%)			0.023
Stage I–II	73 (63.5%)	89 (78.1%)	
Stage III–IV	42 (36.5%)	25 (21.9%)	
Grade, *n* (%)			0.003
G1–2	53 (46.1%)	76 (66.7%)	
G3–4	62 (53.9%)	38 (33.3%)	
Status, *n* (%)			<0.001
Alive	64 (55.7%)	92 (80.7%)	
Dead	51 (44.3%)	22 (19.3%)	

**Table 3:** Univariate and multivariate Cox regression analysis of clinical characteristics.

Characteristic	Univariable		Multivariable	
	HR (95%CI)	FDR	HR (95%CI)	FDR
Age (<60 vs. ≥ 60)	1.206 (0.761–1.911)	0.4863		
Gender (FEMALE vs. MALE)	0.750 (0.468–1.204)	0.3745		
Grade (G1–2 vs. G3–4)	1.073 (0.675–1.704)	0.7666		
Stage (stage I–II vs. stage III–IV)	2.974 (1.874–4.720)	<0.001	1.354 (0.184–9.947)	0.7659
T stage (T1–2 vs. T3–4)	2.991 (1.884–4.749)	<0.001	1.707 (0.231–12.618)	0.7659
N stage (N0 vs. N1)	2.123 (0.518–8.695)	0.3937		
M stage (M0 vs. M1)	4.055 (1.270–12.947)	0.0363	2.890 (0.866–9.644)	0.1688
Risk score	57.037 (16.725–194.506)	<0.001	41.050 (11.493–146.614)	<0.001

**Table 4:** Molecular docking results of DHM with 3 target proteins.

Target	PDB ID	Refinement Resolution (Å)	Affinity (kcal/mol)
DTYMK	1NN0	1.60	−8.1
MAPT	6PXR	1.556	−7.7
UCK2	7SQL	2.40	−8.2

## Data Availability

The data analyzed in the study are available from the following databases: TCGA (https://portal.gdc.cancer.gov/, accessed on 25 November 2024); HCCDB2.0 (http://lifeome.net:809/#/home, accessed on 25 November 2024); PubChem database (https://pubchem.ncbi.nlm.nih.gov/, accessed on 25 November 2024); Swiss Target Prediction (http://swisstargetprediction.ch/, accessed on 25 November 2024); PharmMapper (https://lilab-ecust.cn/pharmmapper/index.html, accessed on 25 November 2024); UniProt (https://www.uniprot.org/, accessed on 25 November 2024); TIP (http://biocc.hrbmu.edu.cn/TIP/, accessed on 25 November 2024); TIDE (http://tide.dfci.harvard.edu/, accessed on 25 November 2024); TISIDB (http://cis.hku.hk/TISIDB/, accessed on 25 November 2024); GDSC (https://www.cancerrxgene.org/, accessed on 25 November 2024); GEO (https://www.ncbi.nlm.nih.gov/geo/); RCSB PDB (https://www.rcsb.org/, accessed on 25 November 2024). Processed data and code may be obtained from the corresponding author upon reasonable request.

## Supplementary Materials

The following supporting information can be downloaded at: https://www.mdpi.com/article/10.3390/cimb47121010/s1.

## Abbreviations

The following abbreviations are used in this manuscript:

AUC	Area Under Curve
CI	Confidence Intervals
DEGs	Differentially Expressed Genes
DHM	Dihydromyricetin
DHMGs	7 DHM-related genes
FDR	False Discovery Rate
GDSC	The Genomics of Drug Sensitivity in Cancer
GEO	Gene Expression Omnibus
GSVA	Gene Set Variation Analysis
HCC	Hepatocellular carcinoma
HR	Hazard Ratio
LASSO	Least Absolute Shrinkage And Selection Operator
MSI Expr Sig	Microsatellite Instability Expression Signature
OS	Overall Survival
RNA-Seq	RNA sequencing
ROC	Receiver Operating Characteristic
SMILES	Simplified molecular input line entry system
SRGs	Sorafenib Resistance Genes
TCGA	The Cancer Genome Atlas Program
TIDE	Tumor Immune Dysfunction and Exclusion
TIP	Tracking Tumor Immunophenotype
