# Supplementary material for: Dihydromyricetin Remodels the Tumor Immune Microenvironment in Hepatocellular Carcinoma: Development and Validation of a Prognostic Model
Source: Curr Issues Mol Biol. 2025 Dec 2;47(12):1010. doi: 10.3390/cimb47121010 (PMC12732133; doi:10.3390/cimb47121010)
Supplement: Supplementary file 1 [file cimb-47-01010-s001.zip › Supplementary/Supplementary figure.pdf]

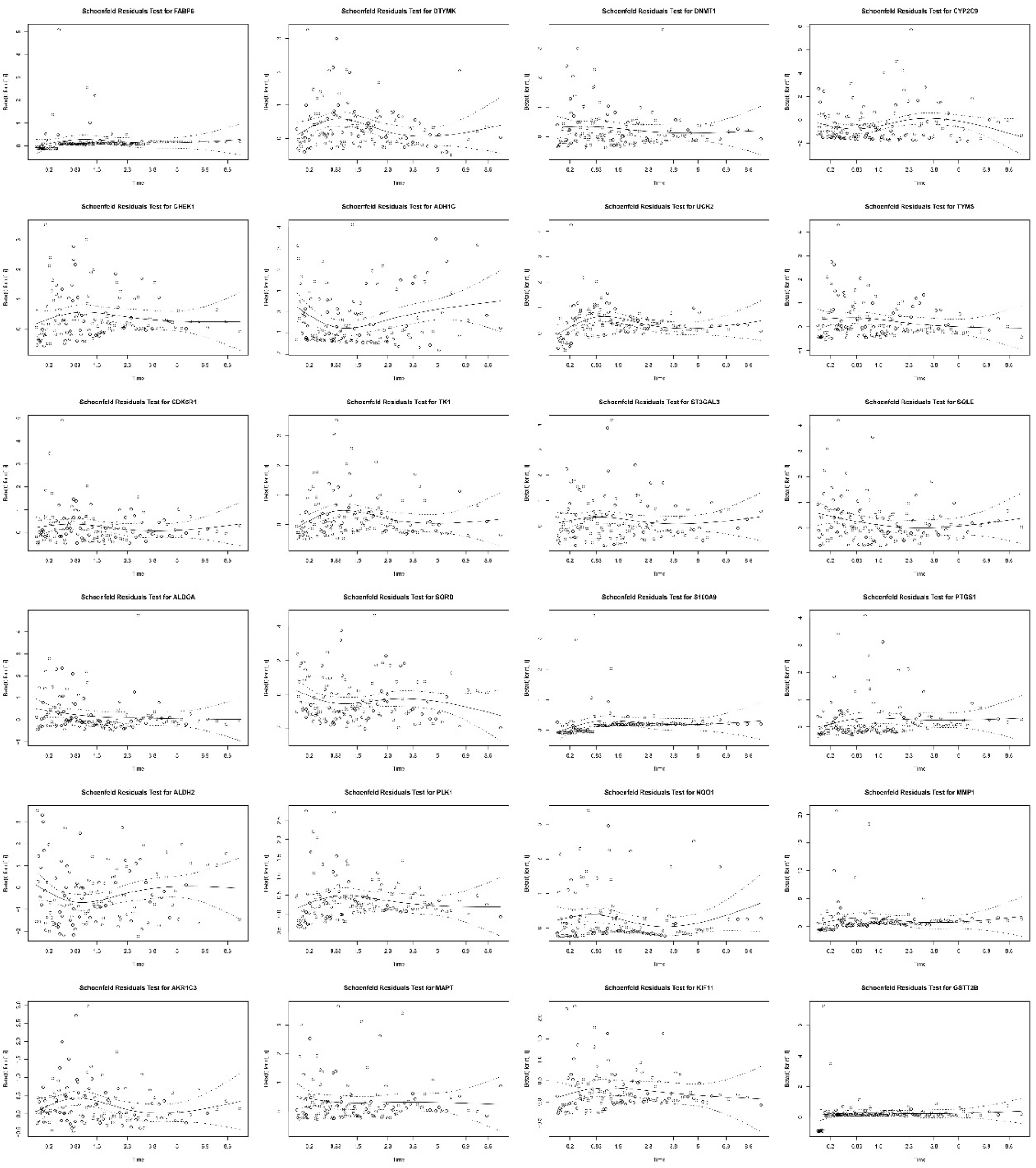

Figure S1 Schoenfeld residual tests for 24 DHM-associated prognostic genes.

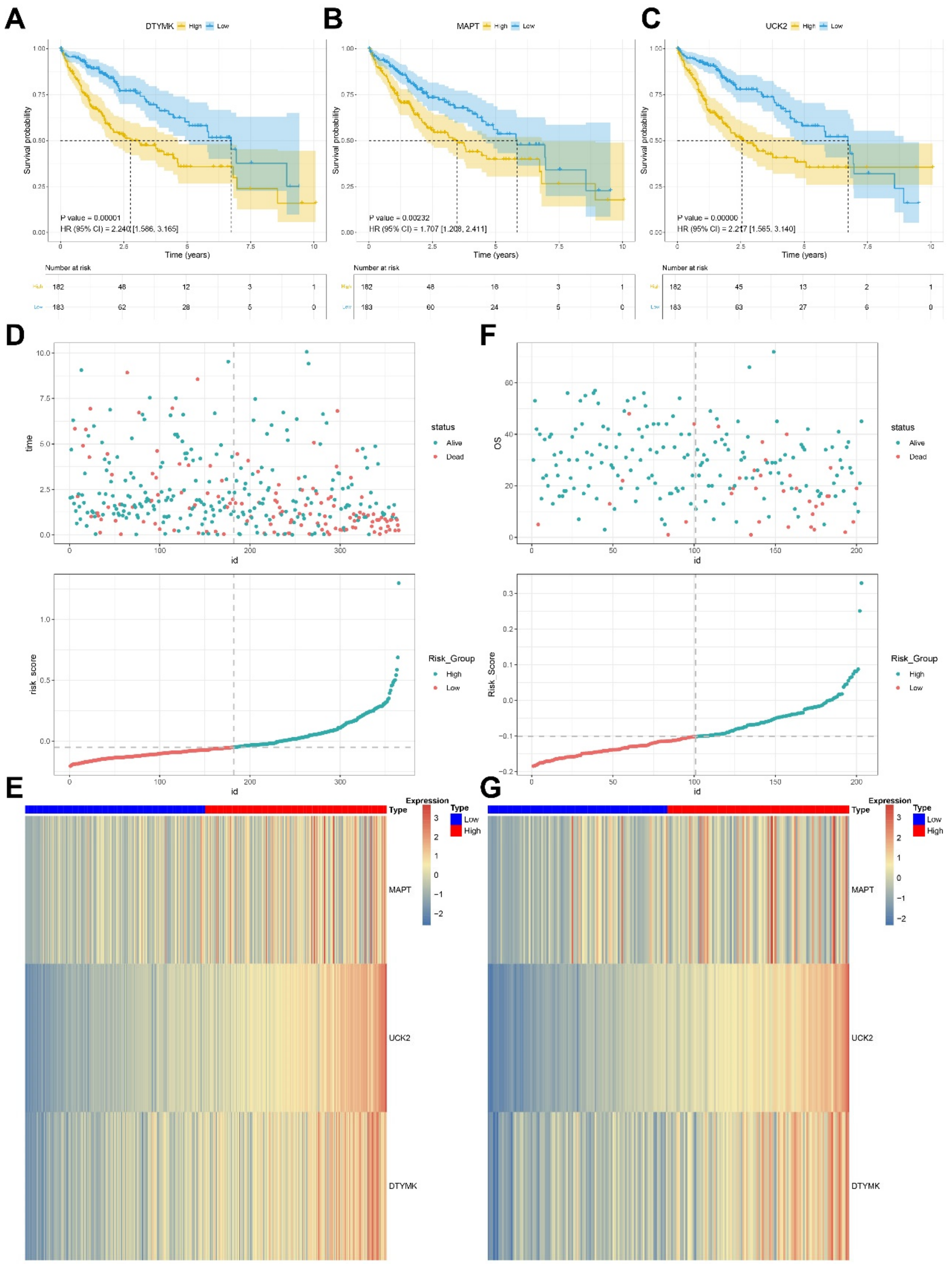

Figure S2 Risk score grouping and model gene expression. (A-C) Survival analysis of DHMGs. (A) DTYMK. (B) MAPT. (C) UCK2. (D)Risk score grouping in TCGA-LIHC. (E) Heatmap of the expression of DHMGs between different risk groups in TCGA-LIHC. (F)Risk score grouping in HCCDB18. (G) Heatmap of the expression of DHMGs between different risk groups in HCCDB18.

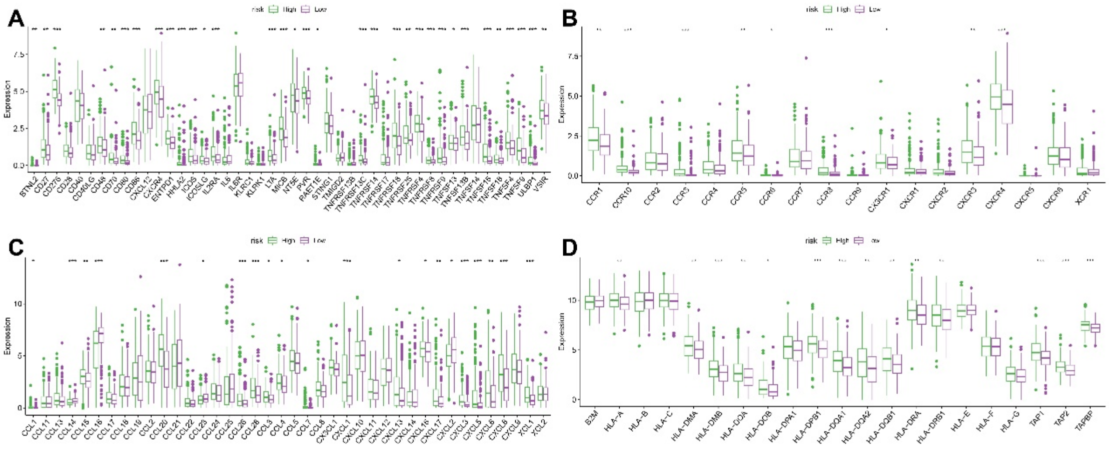

Figure S3 Differential analysis of immune-related factors across different expression groups in the TCGA-LIHC dataset. (A) Immune stimulators. (B) Chemokine receptors. (C) Chemokines. (D) MHC molecules.

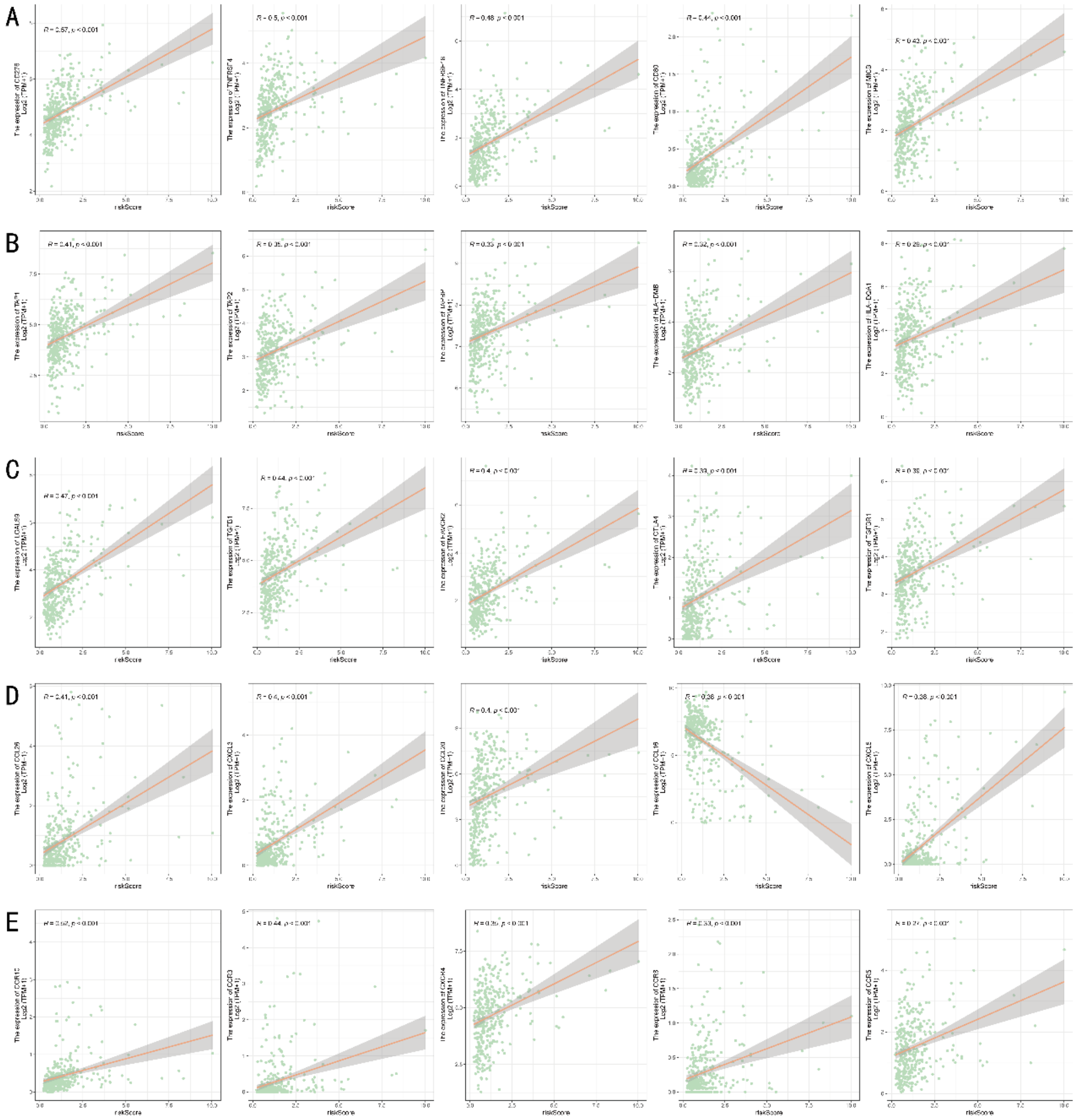

Figure S4 Correlation between risk scores and immune microenvironment factors. (A) Immunostimulators. (B) MHC molecules. (C) Immunoinhibitors. (D) Chemokines. (E) Receptors. (*p<0.05, **p<0.01, ***p<0.001).

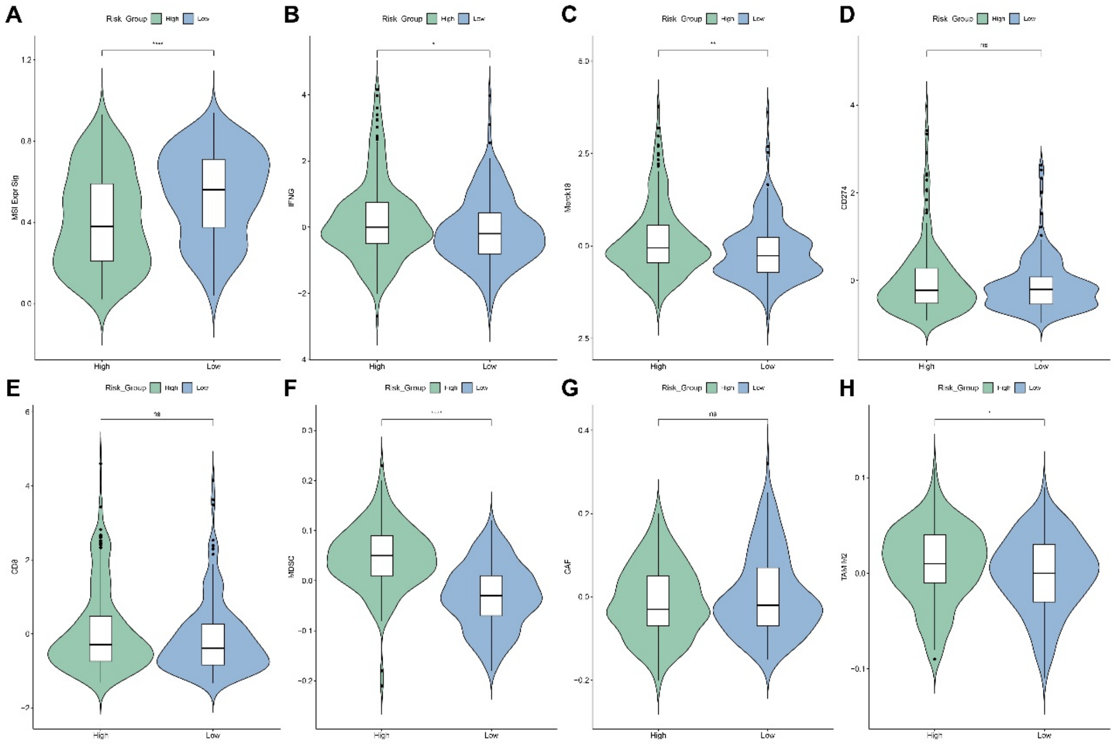

Figure S5 Relationship between different risk groups and TIDE score. (A)MSI Expr Sig. (B) Interferon gamma (IFNG). (C) Merck18. (D) CD274. (E) CD8. (F) Myeloid-derived suppressor cells (MDSC), (G) Tumor-associated fibroblasts (CAF), (H) M2 type tumor-associated macrophages (TAM M2). (*p<0.05, **p<0.01, ***p<0.001, ****p<0.0001, and ns, no statistical difference).

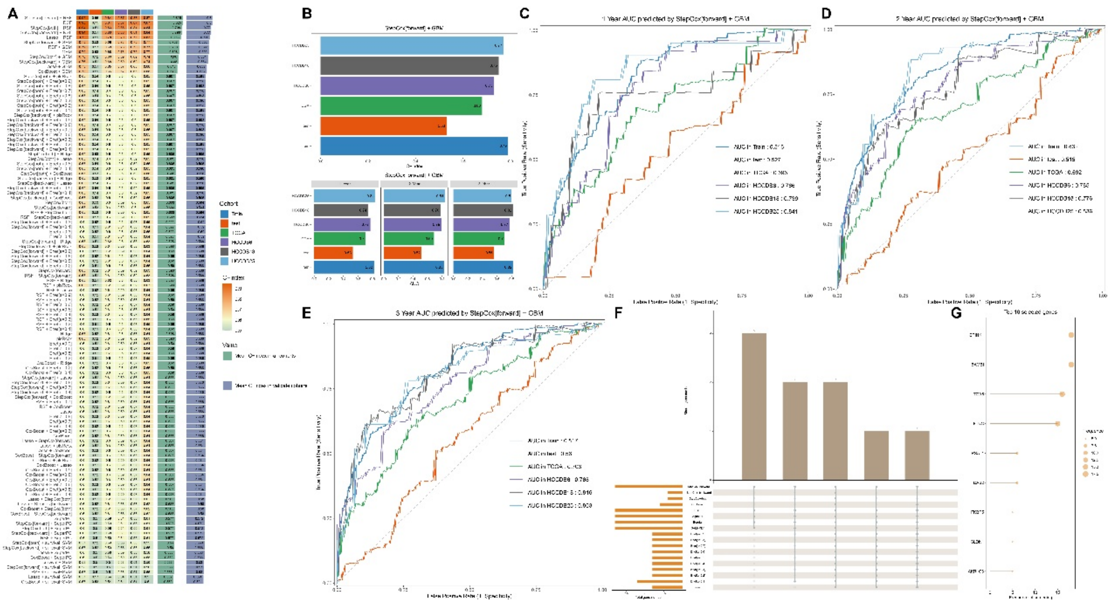

Figure S6 Construction of sorafenib resistance scores and screening of related characterized genes. (A) C-index of 101 machine learning constructed models. (B) AUC of 101 machine learning constructed models. (C) C-index and AUC of the models included in the study. (E-F) The 15 sorafenib resistance-related feature genes screened.

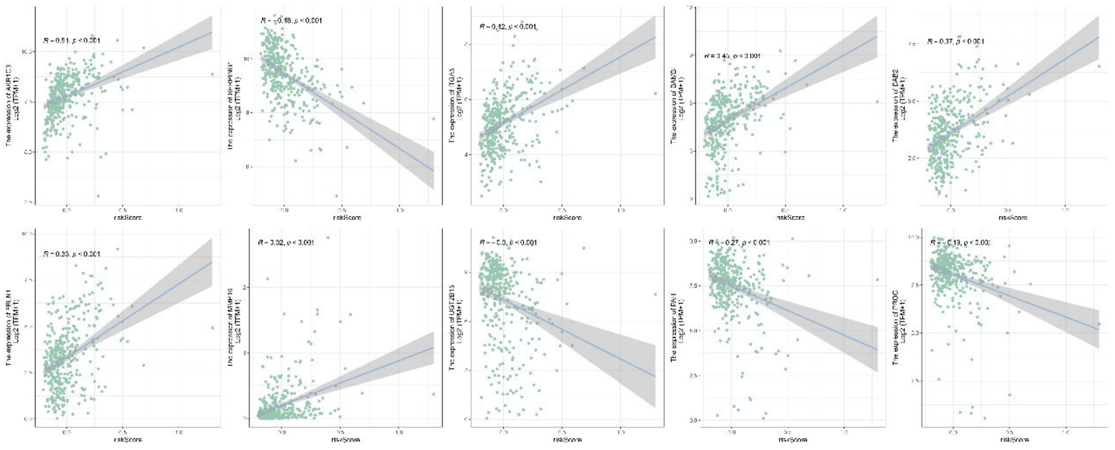

Figure S7 Correlation analysis between risk scores and genes associated with sorafenib resistance.
