# Supplementary figures and images for: Dihydromyricetin Remodels the Tumor Immune Microenvironment in Hepatocellular Carcinoma: Development and Validation of a Prognostic Model
Source: Curr Issues Mol Biol. 2025 Dec 2;47(12):1010. doi: 10.3390/cimb47121010 (PMC12732133; doi:10.3390/cimb47121010)

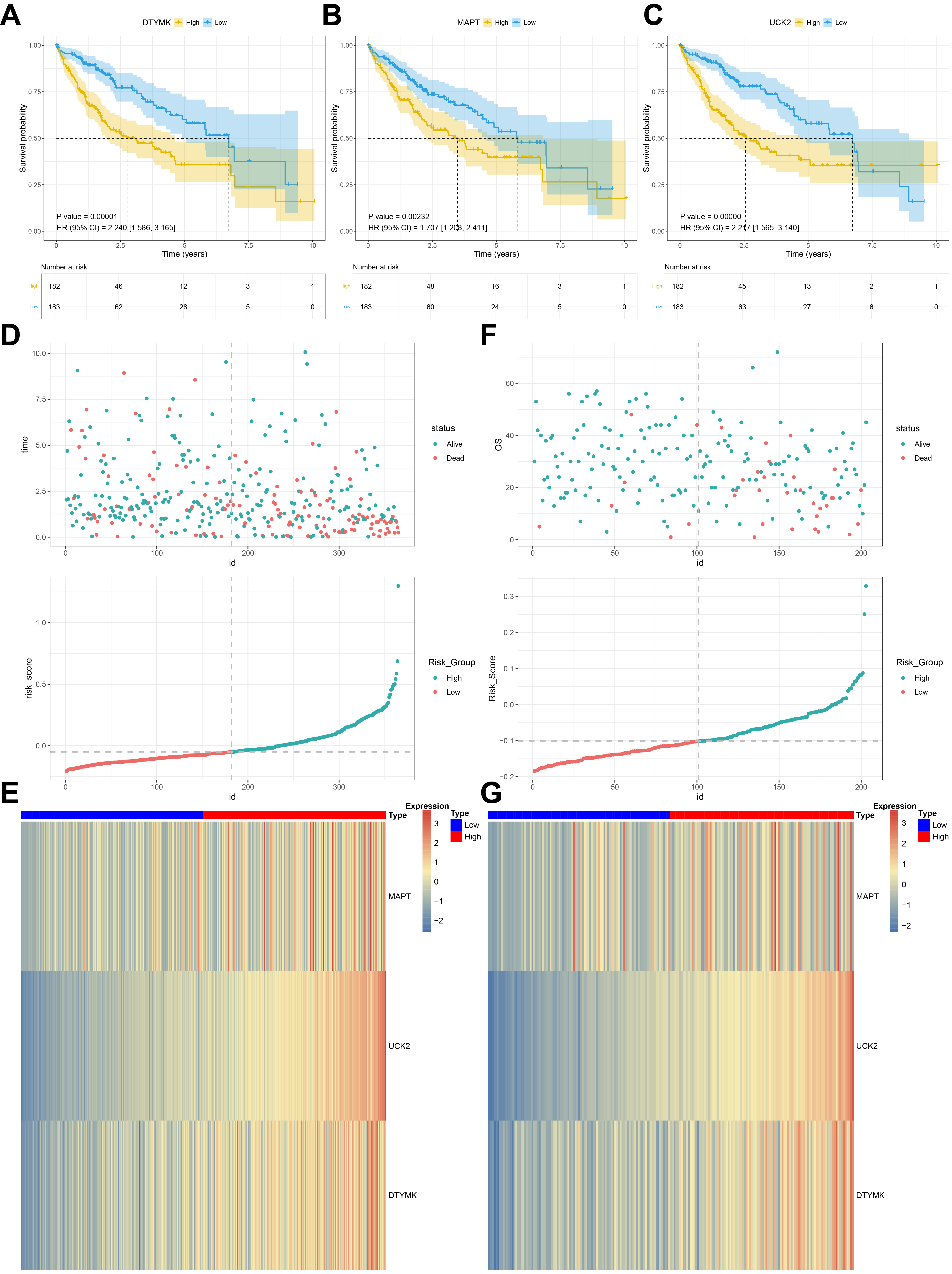

Supplement: Supplementary file 1 [file cimb-47-01010-s001.zip › Supplementary/Supplementary Figure 2.jpg]

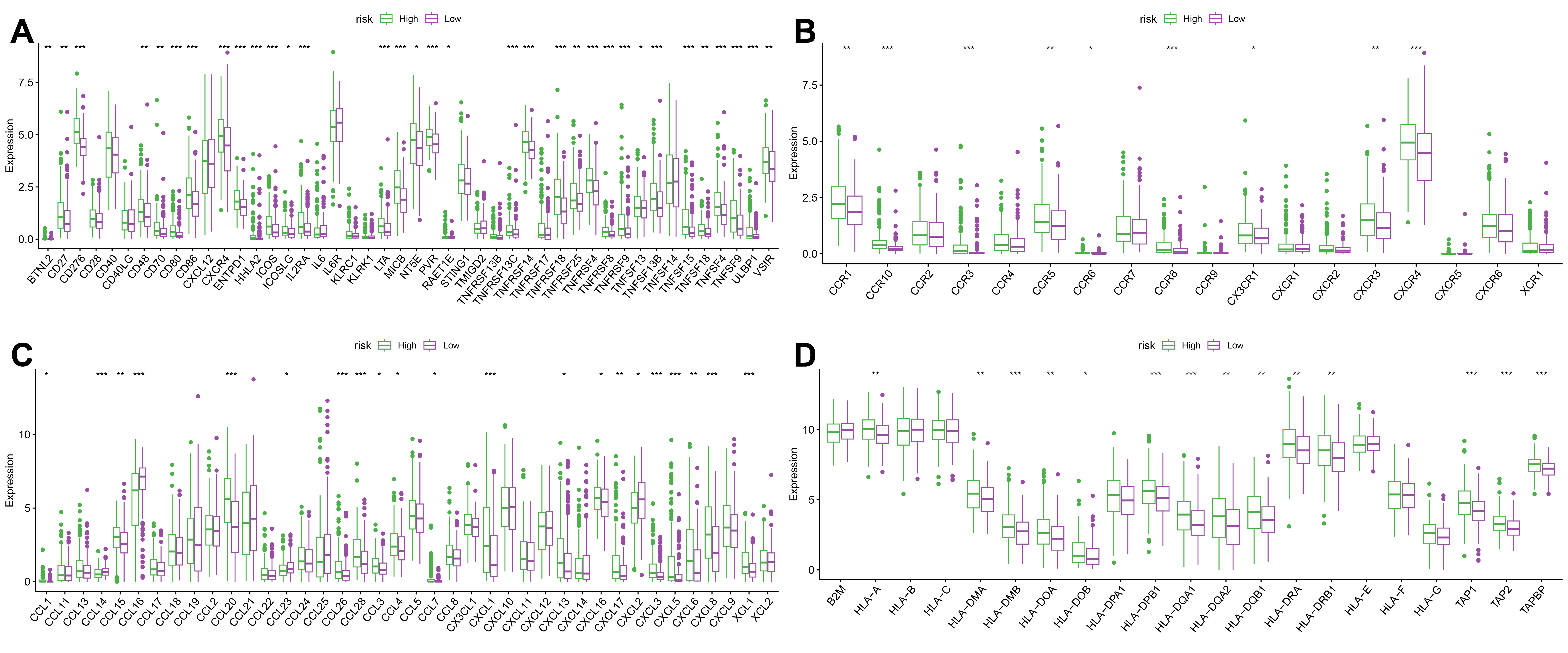

Supplement: Supplementary file 1 [file cimb-47-01010-s001.zip › Supplementary/Supplementary Figure 3.jpg]

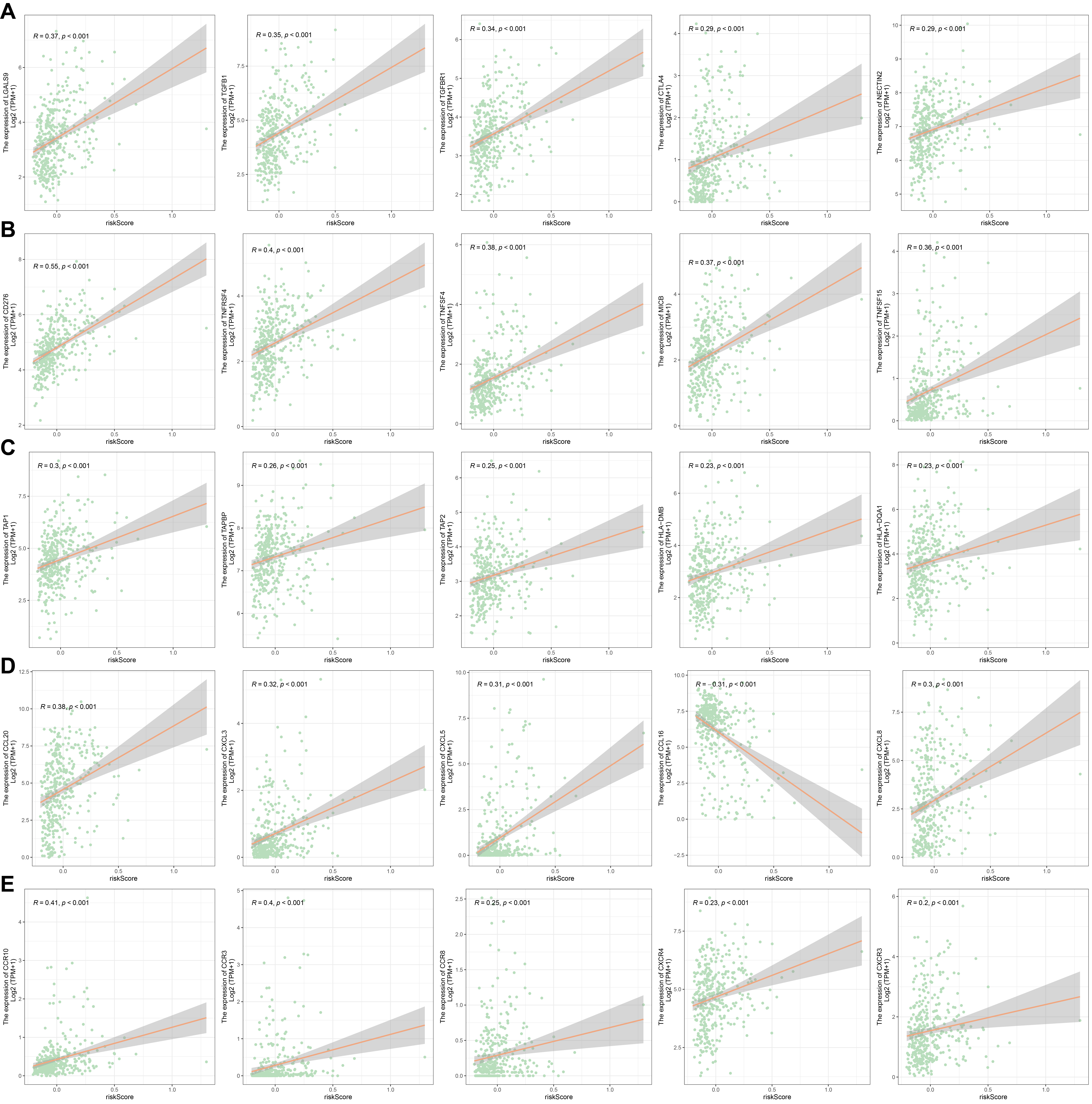

Supplement: Supplementary file 1 [file cimb-47-01010-s001.zip › Supplementary/Supplementary Figure 4.jpg]

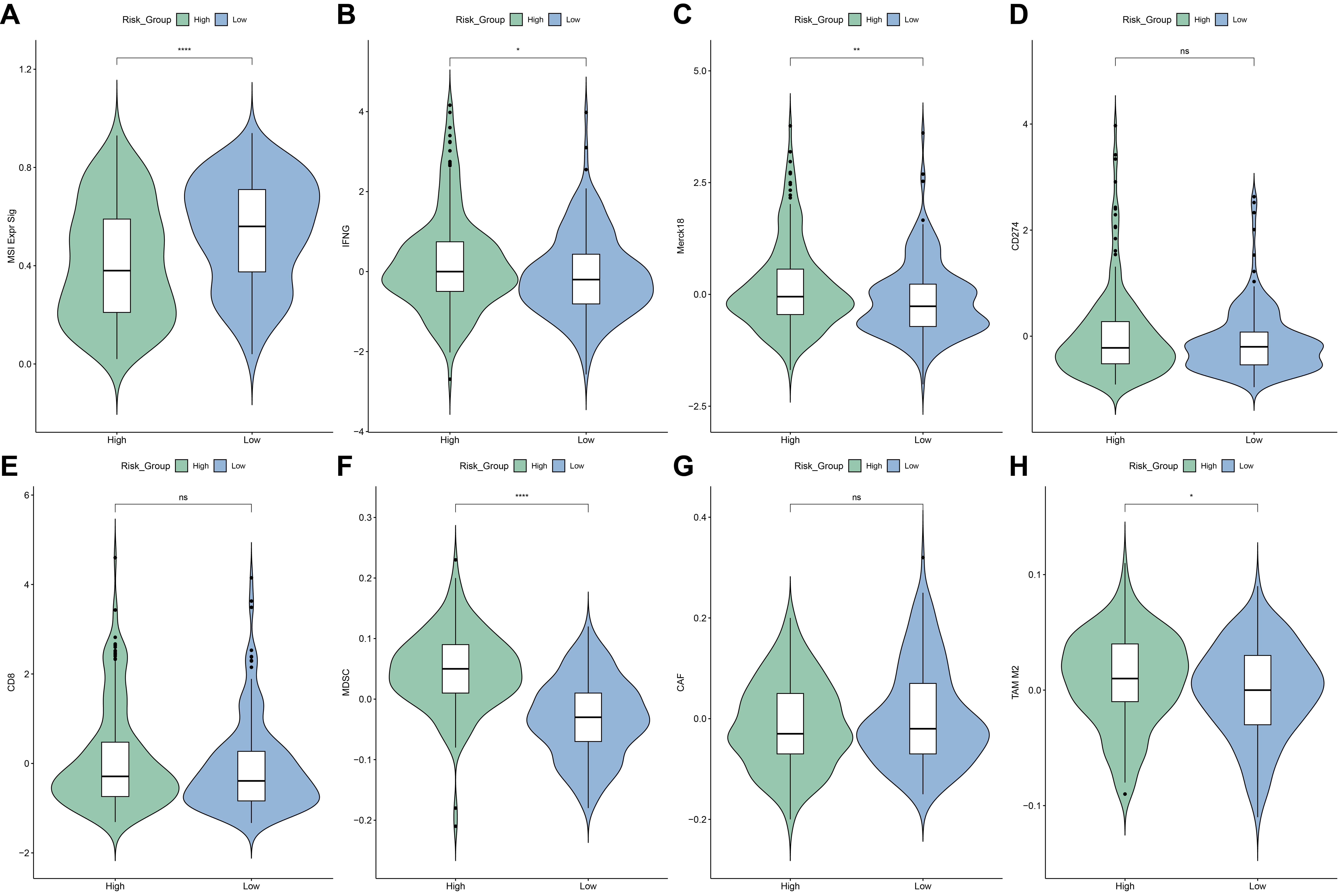

Supplement: Supplementary file 1 [file cimb-47-01010-s001.zip › Supplementary/Supplementary Figure 5.jpg]

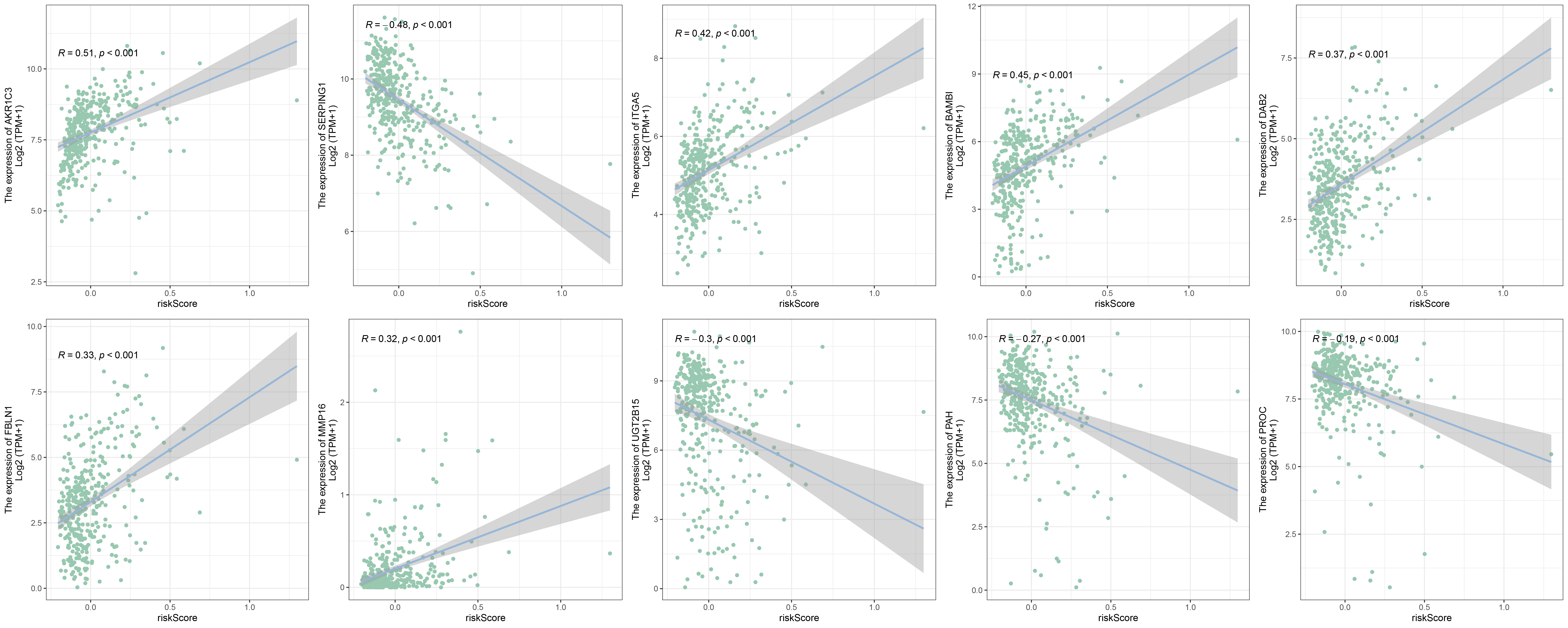

Supplement: Supplementary file 1 [file cimb-47-01010-s001.zip › Supplementary/Supplementary Figure 7.jpg]
